# Becoming Bedridden and Being Bedridden: Implications for Nursing and Care for Older People in Long‐Term Care: A Scoping Review

**DOI:** 10.1111/opn.70015

**Published:** 2025-02-13

**Authors:** Bianca Berger, Fabian Graeb, Manfred Baumann, Reinhold Wolke

**Affiliations:** ^1^ Hochschule Esslingen ‐ Campus Flandernstrasse Esslingen Baden‐Württemberg Germany; ^2^ Hospice Rems‐Murr‐Kreis E. V. Backnang Germany

**Keywords:** bedriddenness, living in bed, local confinement

## Abstract

**Background:**

An increasing number of individuals aged 60 and older will rely on healthcare services, particularly due to increased physical limitations and mobility restrictions. This implies an increase in permanently immobile, often bedridden people who require targeted care. Mobility impairments progress gradually, leading to loss of autonomy and self‐care skills, physical decline, functional deterioration, disability, etc. This review synthesises research findings on the implications of becoming and being bedridden for nursing care of older people in long‐term care.

**Objectives:**

The aim of this scoping review was to explore the current state of research on the topic of bedriddenness in older people in the context of long‐term care and to identify research gaps.

**Methods:**

We analysed English and German language peer‐reviewed publications from the electronic databases MEDLINE (PubMed), CINAHL, LIVIO and Scopus. Publications from 1998 to 2023 were included if they addressed ‘bedriddenness’, the ‘process of becoming bedridden’, ‘prevention’ and the ‘consequences of bedriddenness’, and the ‘care of bedridden people’. The PRISMA‐ScR guidelines were used.

**Results:**

We identified 250 publications based on the defined inclusion criteria. We included 47 in detail condensing their content and organising them according to developed categories, bedriddenness and its prevention, development, consequences and care, which were the subject of intensive research. Although researchers have focused on risk factors for the development of immobility and its negative consequences for those affected, they paid little attention to self‐determination and the organisation of life in bed, which enables those affected to participate. Different ideas regarding the term ‘bedriddenness’ indicate the need for a consensus definition.

**Conclusion:**

Our review shows that articles strongly focus on the process of becoming bedridden. Many publications examine bedridden people's care by emphasising the perspective of (informal) caregivers and the challenges they face. The perspective of bedridden people, together with the consideration of psychosocial dimensions of bedriddenness and the promotion of opportunities for participation, should be focused on in further research. Appropriate concepts for training and nursing practice would be helpful.

**Implications for Practice:**

Bedriddenness is a phenomenon that mainly affects older people, particularly those living in nursing homes. Concepts should be developed that reflect the reality of bedridden people's lives. Targeted interventions to improve the mobility of people with severe mobility impairments are important. Therefore, it is essential to establish educational modules for (informal) caregivers that consider mobility and rehabilitative practices as an integral part of care.

**Summary**

- What does this research add to existing knowledge in gerontology?
  ○ Researchers' findings focus mainly on physical aspects, whereas psychosocial effects are given less attention.
  ○ Participation in, involvement in and the organisation of life in bed need to receive more attention in research, discussion and care practice.
  ○ Different ideas regarding the term ‘bedriddenness’ and the ‘levels of bedriddenness’ indicate the need for a consensus definition.
- What are the implications of this new knowledge for nursing care for and with older adults?
  ○ There is a need for organising the ‘living‐space bed’ and promoting the participation and involvement of bedridden individuals.
  ○ Individualised concepts should be developed that reflect the reality of bedridden people's lives in bed and can be easily integrated into daily routines.
  ○ Room design, engaging activities and meaningful interactions with care staff are crucial.
- How could the findings be used to influence practice, education, research, and policy?
  ○ Care of bedridden individuals at home is important, with family caregivers playing a key role. It is vital to both increase such carers' knowledge and provide them with more support.
  ○ Researchers should consider strategies to improve participation among bedridden people.
  ○ Disciplines, such as nursing science, should focus on preventing immobility and delaying mobility restrictions. This includes creating and implementing multimodal exercise programmes that enhance daily skills.

## 1 Introduction

Mobility restrictions affect 35% of individuals over 70 years and the majority of people aged over 85 years. They are associated with an increased risk of falls, increased hospitalisation, reduced quality of life and even increased mortality. Older people are therefore particularly affected by mobility impairments and their consequences (Freiberger et al. 2020).

If mobility impairments arise, they can gradually lead to bedriddenness. This process of gradual confinement to one place, first described in detail by Zegelin (2005), is accompanied by a reduction in the space for action and possibilities for movement. Initially, the affected person is increasingly reliant on one place—first the home, for example, then a chair, a wheelchair and finally a bed. A reduction in the radius of movement can be accompanied by a loss of self‐determination and self‐care. In the final phase of the process, as a severe form of bedriddenness, the affected person is lying down permanently, ‘around the clock’ (Zegelin 2010). As people adapt their needs to their situation and do not want to be a burden, the bed becomes their living space. Their participation in the community decreases and increasing deprivation determines their everyday life (Berger and Reuther 2022). The affected person's self‐determination in the way they live their life drops severely, and they become dependent on nursing support.

Bedriddenness can physically result in extensive physical deterioration, including a loss of 10%–15% of muscle strength and mass after just 1 week of lying down (Guedes et al. 2018). In hospitalised older people, increasing immobilisation can lead to the development of pressure ulcers, which often contribute to functional decline and disability. Other possible complications include pneumonia, structural changes to joints, contractures, bone loss, orthostatic hypotension, constipation, disturbed sleep patterns and skin damage (Fox et al. 2009). Studies have shown that prolonged bed rest detrimentally affects almost every organ system. Even relatively short episodes of bed rest make older people vulnerable to negative functional developments. Bedriddenness is therefore associated with numerous physical, psychological and social consequences (Berger and Reuther 2022).

Finally, it can be observed that there is an important contextual factor for bedriddenness. Although bedriddenness can affect people in all care settings, those living in nursing homes are more likely to be affected. Around 30%–60% of nursing home residents experience a gradual decline in mobility within 6 months after moving into the nursing home, leading to bedriddenness (DNQP 2014). According to Schrank et al. (2013), the prevalence of bedriddenness in nursing homes is close to 50%.

As bedriddenness is a phenomenon of great importance for nursing care, it is necessary to get an overview of the research. This scoping review outlines the results of research in the period 1998–2023 on becoming and being bedridden and the implications for nursing care for older people in long‐term care.

## 2 Research Question and Aim

The aim of this scoping review was to explore the current state of research on the topic of bedriddenness in older people in long‐term care and to identify research gaps for further research. We focused on the phenomenon of bedriddenness, the process of becoming bedridden, the prevention of bedriddenness, the consequences of bedriddenness and the care of bedridden persons. We also examined whether care concepts exist that also consider the social and psychological needs of those affected and that involve those affected in the organisation of their lives (in bed), in addition to the physical aspects. The specific population was individuals aged 65 years and older who were in long‐term care.

We undertook this project to systematise the content and results of a wide range of existing articles. In addition, we aimed to present nursing implications for the care of older people and outline areas for further research.

## 3 Material and Methods

In order to explore the extent, range and nature of the phenomenon in research; identify unaddressed aspects; and thereby highlight gaps in the literature, we conducted a scoping review based on the methodology proposed by Arksey and O'Malley (2005) and the methodological framework further developed by Levac et al. (2010) and the Joanna Briggs Institute (JBI) (Peters et al. 2020; Tricco et al. 2018).

Scoping reviews differ from systematic reviews because they provide a broad overview, regardless of the quality of the respective studies, publications or evidence. In cases of bedriddenness, this approach is helpful for illuminating new perspectives, clarifying key concepts or identifying research gaps (Peters et al. 2020), rather than merely pinpointing the efficacy of interventions. We refrained from conducting a critical appraisal or assessing the methodological quality of the reviewed publications because our aim was to present a broad overview of the topic ‘becoming or being bedridden in older people in long‐term care’. We adhered to the Preferred Reporting Items for Systematic Reviews and Meta‐analysis Extension for Scoping Reviews (PRISMA‐ScR) guidelines (Tricco et al. 2018); (Peters et al. 2020).

We conducted this scoping review in five steps (Arksey and O'Malley 2005; Levac et al. 2010)

– Identification of the research question and aim.
– Identification of relevant studies.
– Selection of studies.
– Data collection and extraction.
– Compilation, summarisation and reporting of the results.

### 3.1 Identification of the Research Question and Aim

The aim of the scoping review and the research question have already been described under the ‘Research question and aim’ section.

### 3.2 Identification of Relevant Studies

Initially, we conducted a limited search in two databases, followed by an analysis of the discovered works with regard to the text terms and keywords. We also verified whether scoping reviews on this topic already existed and, if so, what content they covered and the search strategies they applied. We identified three such reviews. We then adjusted the research questions, keywords and search strategy and discussed inclusion and exclusion criteria in the final consultation. We included and analysed English and German language peer‐reviewed publications in the field of long‐term care if they addressed the following topics: ‘bedriddenness’, the ‘process of becoming bedridden’, ‘prevention adverse outcomes of bedriddenness’, the ‘consequences of bedriddenness’ and the ‘care of bedridden older people’ (aged 65 and older). We used the electronic databases MEDLINE (PubMed), CINAHL, LIVIO and Scopus from January to April 2023. Data S3 shows the search strings for the MEDLINE (PubMed) database. This search strategy was adapted for the remaining databases.

### 3.3 Selection of Studies

#### 3.3.1 Title and Abstract Screening

Two of the authors conducted the title and abstract screening using the systematic review software Covidence, discussed different assessments and obtained a third opinion when necessary. We excluded the following publications from the review: conference abstracts, posters, brief overviews, editorials and commentaries or letters to the editor.

#### 3.3.2 Full‐Text Screening

Two of the authors procured and evaluated the full texts in a blinded process and documented the reason behind any exclusion, once again using the Covidence systematic review tool. Once this twofold review was complete, we analysed any conflicting evaluations, analysing our differing assessments and resolving the divergences through discussion. No third‐party involvement proved necessary. As our aim was to present the breadth of the discourse on the topic of bedridden people, we did not undertake a critical appraisal of individual sources of evidence.

### 3.4 Data Collection and Extraction

Of the articles collected, we descriptively extracted the following information: author, objective, year of publication, study design, sample, study setting country and category (see Data S1). Although these categories were already evident in our search strategy, a more in‐depth review of the publications was necessary to refine and complete the content delineation. Each category was further defined in detail, and the assignment of texts was re‐evaluated according to these definitions.

For the synthesis, we conducted a qualitative synthesis that briefly outlines the results to illuminate the breadth of the discourse. The search strategy is documented in a flow chart provided in the Data S2. Filling in each category for the publications allowed us to present our results in a structured manner.

## 4 Results

A database search identified 1059 articles, whereas a manual search identified 7 additional articles for 1066. After removing duplicates, 650 articles with regard to titles and abstracts were included for screening. We then conducted full‐text screening for 249 papers, which we assessed further until we could retrieve 47 papers that met the criteria for this scoping review. The results of this comprehensive search process are presented in a flow chart (see Data S2).

**FIGURE 1:**
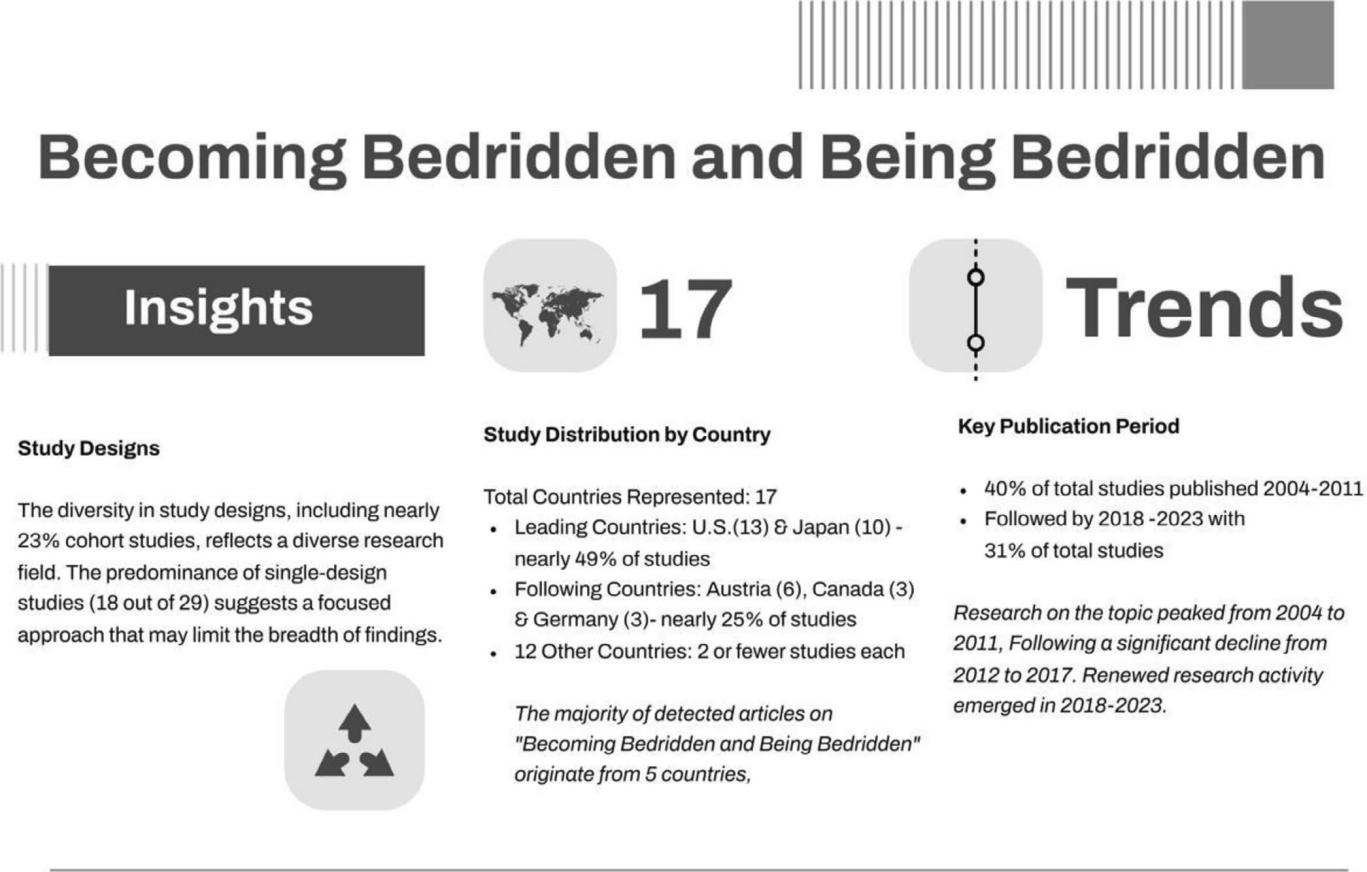
Scoping review insights: study design, study distribution by country and year.

Most of the publications came from the United States (13), followed by Japan (10) and Austria (6). Germany and Canada had three publications each (Figure 1). Most of the publications (approx. 60%) were characterised by a quantitative study design. Further details, including a representation of publications by year, can be found in Figure 1. The publications were categorised as ‘process of becoming bedridden’ (16), ‘care of bedridden people’ (14), ‘adverse outcome and consequences of bedriddenness, their treatment and prevention’ (9) and ‘prevention of bedriddenness’ (8) (Data S1).

In some publications, long‐term care is not explicitly mentioned, but the theoretical findings on the terms and phrases related to bedriddenness are relevant to the field of long‐term care.

### 4.1 Process of Becoming Bedridden

According to Zegelin's concept of ‘local confinement’, becoming bedridden is a process. Her findings indicate that bedriddenness develops gradually and can be understood as a process that includes various distinct phases.

The process begins with a sense of insecurity when moving, for example, when walking, standing up and sitting down or climbing stairs. Those affected experience this as a ‘state of instability’. They limit their range of movement to their own home because of a fear of falls, require significant support for daily activities and consider the use of assistive devices.

The next phase is an ‘event’ phase, where a triggering factor, such as a fall, occurs, leading to further restrictions in mobility, often confining individuals to a single room. Hospital stays can exacerbate this situation, as the bed becomes the central location, contributing to muscle atrophy and reinforcing the fear of falls, which further limits mobility.

In the subsequent phase, ‘immobility in the space’, individuals spend most of their time in a fixed location, requiring increasing assistance and experiencing health complications.

The ‘local confinement’ phase is characterised by difficulties in transferring between the bed and other furniture, severely limiting mobility options.

Ultimately, the final phase culminates in ‘bedriddenness', where individuals hardly leave the bed, which then becomes their primary living space, significantly increasing their sense of dependence.

Zegelin also names various influencing factors, including the affected person's individual aspects, their interactions and structural influences, such as the time caregivers are exposed to pressure. She presents many of these factors as modifiable, that is, the involuntary fate of being bedridden could be avoided. For example, Zegelin recommends creating opportunities for movement that the person in need of care can find meaningful (Zegelin 2005, 2008).

Schirghuber's research group has conducted concept analyses to concretise and develop terms that differentiate between phases in the process of becoming bedridden. For example, the researchers distinguish between ‘chair‐bound’, ‘bed‐bound’ and ‘house‐bound’. However, these newly developed concepts have not yet been applied (Schirghuber and Schrems 2018, 2021, 2023).

Bedriddenness is a central issue in nursing homes. A study on inpatient long‐term care in Austria with almost 3000 participants revealed a prevalence rate of 49.8% (Schrank et al. 2013). Staffing level is the strongest predictor of time spent in bed. It refers to the number of nursing and caregiving staff available per resident in a nursing home. Residents in facilities with lower staffing levels are six times more likely to spend over 50% of their time in bed (Bates‐Jensen, Schnelle, et al. 2004). This indicates that better staff ratios may lead to increased engagement and activity among residents, which in turn reduces excessive in‐bed time and is closely related to the process of becoming bedridden. Bates‐Jensen, Alessi, et al. (2004) pointed out that homes with a higher number of bedridden people do not offer fewer activity or mobility programmes than those with a lower number. Moreover, the number of bedridden individuals is often underestimated by the staff of these facilities.

The ability to perform activities of daily living (ADL) independently is an important indicator of a person's level of independence. Difficulty in low‐difficulty activities, such as eating independently, going to the toilet and moving in bed, can indicate that the individual is becoming bedridden. The process often begins with difficulties in activities at a higher level, such as putting on trousers, getting up from a seated position, climbing stairs and washing the whole body. According to Sato et al. (2001), struggles with low‐difficulty tasks—like eating and toileting—can signal an individual's declining functional status, increasing the risk of bedriddenness. Monitoring ADL performance is therefore essential for timely interventions to maintain autonomy. Understanding these levels is crucial for assessing and supporting individuals at risk of further decline.

For people who live at home, longer periods of bed rest lead to an increased need for assistance with everyday activities. Interestingly, this effect is more pronounced in people who have no walking difficulties (Gill et al. 2004). Prolonged bed rest should be avoided to ensure functional independence (Gill et al. 2015).

However, there are also situations in which those affected may see good reasons to stay in bed, such as illness, limited mobility and tiredness. They can use the time to conserve energy and prepare for future activities. Some perceive staying in bed as a form of partial autonomy that gives them control over their living space. However, this can also lead to a decline in health and increased dependency (Fox et al. 2009).

However, approximately 3–5 months before death, the number of days in bed and the burden of bed rest increase significantly, and going to bed is probably an indicator of imminent death (Gill et al. 2018, 2019).

### 4.2 Care of Bedridden Persons

Providing care to bedridden individuals is a complex task that presents substantial challenges for both family members and professionals. Various factors influence the burden on caregivers, including their own health and a bedridden individual's level of dependency (Bekdemir and Ilhan 2019).

Relatives caring for older family members with chronic degenerative diseases often experience anxiety and exhaustion. The daily responsibilities of care, coupled with the emotional strain of losing a ‘healthy family member’ and facing mortality, are significant stressors (Campos et al. 2021; Mamom and Daovisan 2022). Informal caregivers are willing to accept support from volunteers, but it is important that they belong to an organisation that takes responsibility for the deployment and quality of help (Abarca et al. 2018). In developing and emerging countries, families bear the primary responsibility for care. This can lead to medical complications if the care provided is inadequate (Bains and Minhas 2011).

Some researchers have proposed ambient‐assisted living solutions as potential means to alleviate the burden of home care. These include aids, such as a mechatronic system to assist with repositioning (Bruno et al. 2016), as well as air‐cell mattresses with an automatic ‘turning option’ (Futamura et al. 2008; Izutsu et al. 1998).

Digital technologies and telemedicine offer bedridden patients access to healthcare services without the need for physical presence. These technologies can help highlight the needs of individuals by providing information, facilitating social interaction, offering emotional support and enabling communication with caregivers (Pinero de Planza et al. 2021).

Although therapies for bedridden patients, such as home massage therapy, have no significant effects (Hirakawa et al. 2005), nursing prophylaxis is crucial to prevent serious consequences. Multi‐professional teamwork is recommended to implement interventions aimed at improving mobility and reducing the frequency and severity of immobility complications (Walsh et al. 1999).

Tube feeding can probably extend a bedridden individual's lifespan by 6 months. If a patient has already been bedridden for more than half a year, the use of a feeding tube might only extend their survival time by another half year. This information could help families in making decisions regarding tube feeding (Kosaka et al. 2012).

Therefore, caring for bedridden people is a complex task. This situation prompts a thorough evaluation of current and future care systems and their implications for nursing care. The focus should be on improving care for bedridden individuals (Imai 1998).

### 4.3 Prevention of Bedriddenness

Older individuals lose muscle mass more rapidly than younger individuals during prolonged periods of inactivity, for example, while recovering from an illness or injury (English and Paddon‐Jones 2010). Atrophy is primarily caused by decreased muscle protein synthesis. Repeated short periods of inactivity (< 10 days) throughout life also contribute significantly to the development of age‐related sarcopenia (Wall et al. 2013).

Physical activity to maintain and promote mobility may therefore help compensate for muscle loss. However, mere activity guidelines, such as 2000 steps per day or 150 min. of moderate exercise per week, are insufficient to completely prevent muscle loss (Arentson‐Lantz et al. 2019). In addition to the process of age‐related physiological degradation, nutrition also plays a crucial role. In order to slow the breakdown, researchers recommend that patients take nutritional supplements, for example, amino acids or proteins, to maintain muscle protein synthesis (Arentson‐Lantz et al. 2019; English and Paddon‐Jones 2010).

Considering these findings, the role of assistive equipment in promoting mobility also becomes important. One study shows that some people still experience difficulties despite the use of mobility aids. These residual difficulties lead to an increased need for personal assistance. However, aids are not equally effective for all users, highlighting the importance of appropriate aid selection and follow‐up assessment (Taylor and Hoenig 2004).

Care facilities that implement a rehabilitative approach have a lower prevalence of bedriddenness and higher participation in social activities (Vähäkangas et al. 2008). This indicates that immobility is influenced by not only illness or age but also individual and environmental factors. The way in which carers perceive their role in terms of their ability to influence the mobility of older people and how they coordinate individual interventions are essential (Reuther 2014). In addition, awareness and knowledge of immobility risks and signs of progressive impairment are central to the implementation of such a rehabilitative approach. For example, the inability to stand up without using one's hands for support can be a sign of progressive impairment. Interventions aimed at improving one's ability to rise from a bed or chair can be used to quantify improvements or declines in ability, as well as signal increasing immobilisation (Alexander et al. 2000). Self‐learning modules, including those on immobility in older people, are therefore an important component in supporting caregivers to initiate strategies to prevent bedriddenness (Fletcher 2005).

### 4.4 Adverse Outcomes and Consequences of Bedriddenness, Their Treatment and Prevention

Bedriddenness can have various physical, psychological and social consequences, leading to a cascade of dependencies and risk factors (Wick 2010). One such example is the link between bed rest and insomnia. Older people who spend 5–7 days in bed experience more insomnia than those who spend less or no time in bed (Fox et al. 2010a). In addition, prolonged bed rest may increase orthostatic intolerance (Fox et al. 2010b) and lead to the loss of postural muscles (Ikezoe et al. 2012).

Other researchers have described bed rest's effects on the cardiovascular system. They found a significantly lower variability in systolic blood pressure over 24 h in bedridden people (Tsuchihashi et al. 2002). An observational study in nursing homes surprisingly found no difference in the incidence of venous thromboembolic events between immobile and (partially) mobile people (Gatt et al. 2004). Skin damage, such as pressure sores, is also associated with bedriddenness (Hampton 2011; Sanada 2001). Other risk factors for skin damage may include a low ankle‐brachial index, prolonged bed rest, male sex (Okuwa et al. 2006), poor nutritional status (Santosa et al. 2020) and bone prominence. In addition, infections and colonisation by *S. aureus* occur more frequently in bedridden patients (Silva et al. 2022).

## 5 Discussion and Conclusion

We analysed the 47 identified studies in relation to the following categories, ‘the process of becoming bedridden’, ‘the care of bedridden people’, ‘the prevention of bedriddenness’, and ‘the adverse outcomes and consequences of bedriddenness’, and identified the following implications for nursing.

Regarding ‘the process of becoming bedridden’, it is crucial that caregivers understand risk factors. This knowledge allows them to detect early signs of local confinement and initiate individual care interventions to maintain mobility and counteract progression. Zegelin's findings are still of central importance because they explain the process and provide caregivers with a useful framework outlining various influencing factors and interventions. Further work by Schirghuber and Schrems (2018, 2021, 2023) could help in refine the terminology and process phases. The results should be validated in further nursing practice studies.

At the same time, it is important for caregivers to understand that those affected see benefits from remaining in bed—for example, they may experience partial autonomy in their bed environment. We could not find any publications that deal with the negotiation processes and examine how the participation of those affected can be promoted in order to balance well‐being and partial autonomy against the negative consequences of remaining in bed. Whether other social factors, such as social isolation, loneliness, excessive demands on relatives and lack of time, could be risk factors for bedrest has not yet been investigated. Bedriddenness continues to be a widespread phenomenon that is often underestimated by those involved. Prevalence studies, including studies on home care, are therefore useful to emphasise its importance.

The category of ‘the care of bedridden people’ has various political and nursing implications. Considering demographic changes, country‐specific strategies for nursing people with bedriddenness and prevention of bedriddenness are essential. As the continued decrease in skilled workers means that family members will continue to provide a large proportion of care services in the future, it is crucial to provide relatives with the skills they need to care for bedridden persons. Family caregivers should be equipped with the necessary skills, and the stressors they face should be addressed. The demand for ambient‐assisted living (AAL) applications will increase, and their ethical use should be analysed.

Caregivers should advocate for the needs of affected individuals and promote the use of digital technologies and telemedicine to improve their participation in health services and society. There is a lack of research focusing on participation and the design of living spaces in bed. It is important to develop specific concepts that are tailored to bedridden people's needs and consider the reality of their lives. This includes elements, such as room design, the development of meaningful activities and fostering interactions with care staff.

In ‘the prevention of bedriddenness’ category, researchers highlighted the need for a multimodal approach to prevent increasing muscle loss that considers nutrition, exercise and rehabilitative care practices. Caregivers should recognise mobility enhancement as an integral part of their work. Therefore, specialised education and training programmes for maintaining and improving mobility are central to providing carers with the necessary skills. This includes recognising and considering the individual attitudes and coping strategies of the affected individuals. However, it is equally vital to ensure adequate staffing levels that make such a method of working possible, emphasising both high‐quality staff and sufficient time capacities to effectively implement these strategies. Research focusing on improving the mobility of people with severe impairments and enabling independent movement is necessary. It is remarkable that the publications we reviewed so rarely addressed the perspectives of those affected. Little work has been done to understand their personal desires and the reasons why they either no longer want to or are no longer able to move.

Finally, the category ‘adverse consequences and effects of bedriddenness’ has the following implications for care: As the physical consequences of bedriddenness are well documented, caregivers should be informed and implement preventive measures. This also includes the assessment of individual risk factors and their continuous monitoring in order to initiate suitable interventions in cooperation with other specialists. However, we have identified a significant gap regarding the psychosocial aspects associated with bedriddenness. Although the physical consequences are well documented, research indicates that psychosocial impacts, such as isolation, depression and anxiety, are often overlooked. This highlights the need for concepts and interventions specifically tailored to address these psychosocial needs.

The key findings of this review are as follows: Although bedriddenness is a phenomenon of great importance for nursing care, the complexity of bedriddenness with regard to its social and psychological implications is hardly considered. Furthermore, the term ‘bedriddenness’ is used vaguely and ambiguously in international contexts (Schirghuber and Schrems 2018), and there are different ideas regarding the ‘levels of bedriddenness’. Although we acknowledge that Schirghuber's research group has developed important distinctions between the terms ‘chair‐bound’, ‘bed‐bound’ and ‘house‐bound’, these frameworks remain unvalidated. We included these terms to emphasise their relevance and potential application to understanding the transition to being bedridden. Future studies could explore the practical implications of these categories in nursing practice, helping to validate their use.

In summary, further research is needed in the field of care for older bedridden patients, particularly regarding the perspectives of those affected and the psychosocial dimensions of bedriddenness. It is essential to highlight the lack of meaning‐oriented research utilising phenomenological and hermeneutical approaches, as well as the need for an existential perspective. Additionally, exploring the meanings of caring, health and well‐being in this context is crucial for developing a more comprehensive understanding of the challenges faced by these individuals and those who care for them.

## 6 Limitations

We did not address findings that guide nursing practice because the grey literature was not reviewed. Our focus was on long‐term care, acknowledging that hospitalisation could be the beginning of local confinement. There is no agreed‐upon definition of ‘bedriddenness’ that underlies the studies. In the publications, terms, such as ‘bed rest’ and ‘bedriddenness’, were used synonymously. Due to our language skills, we only included German and English language articles. Additionally, we limited our review to studies published within the last 25 years. Previous publications were not considered, but they could have provided further insights into this topic.

## Author Contributions

**Bianca Berger:** conceptualisation, data curation, formal analysis, investigation, methodology, project administration, resources, writing – original draft. **Fabian Graeb:** formal analysis, investigation, writing – review and editing. **Manfred Baumann:** formal analysis, investigation, validation, writing – review and editing. **Reinhold Wolke:** writing – review and editing. **Sven Reuther:** research, screening and selection of studies and data extraction.

## Conflicts of Interest

The authors declare no conflicts of interest.

## Supporting information


Data S1

Data S2

Data S3

## Data Availability

As we created no datasets as part of our study, we do not intend to release data for this article.

## References

[opn70015-bib-0001] Abarca, E. , S. Campos , V. Herskovic , and C. Fuentes . 2018. “Perceptions on Technology for Volunteer Respite Care for Bedridden Elders in Chile.” International Journal of Qualitative Studies on Health and Well‐Being 13, no. 1: 1422663. 10.1080/17482631.2017.1422663. 29336722 PMC5769803

[opn70015-bib-0002] Alexander, N. B. , A. T. Galecki , L. V. Nyquist , et al. 2000. “Chair and Bed Rise Performance in ADL‐Impaired Congregate Housing Residents.” Journal of the American Geriatrics Society 48, no. 5: 526–533. 10.1111/j.1532-5415.2000.tb04999.x. 10811546

[opn70015-bib-0003] Arentson‐Lantz, E. , E. Galvan , A. Wacher , C. S. Fry , and D. Paddon‐Jones . 2019. “2,000 Steps/Day Does Not Fully Protect Skeletal Muscle Health in Older Adults During Bed Rest.” Journal of Aging and Physical Activity 27, no. 2: 191–197. 10.1123/japa.2018-0093. 29989486 PMC6710835

[opn70015-bib-0004] Arksey, H. , and L. O'Malley . 2005. “Scoping Studies: Towards a Methodological Framework.” International Journal of Social Research Methodology 8, no. 1: 19–32. 10.1080/1364557032000119616.

[opn70015-bib-0005] Bains, P. , and A. S. Minhas . 2011. “Profile of Home‐Based Caregivers of Bedridden Patients in North India.” Indian Journal of Community Medicine 36, no. 2: 114–119. http://www.redi‐bw.de/db/ebsco.php/search.ebscohost.com/login.aspx%3fdirect%3dtrue%26db%3dcin20%26AN%3d104689123%26site%3dehost‐live. 21976795 10.4103/0970-0218.84129 PMC3180935

[opn70015-bib-0006] Bates‐Jensen, B. M. , C. A. Alessi , M. Cadogan , et al. 2004a. “The Minimum Data Set Bedfast Quality Indicator: Differences Among Nursing Homes.” Nursing Research 53, no. 4: 260–272. 10.1097/00006199-200407000-00009. 15266165

[opn70015-bib-0007] Bates‐Jensen, B. M. , J. F. Schnelle , C. A. Alessi , N. R. Al‐Samarrai , and L. Levy‐Storms . 2004b. “The Effects of Staffing on In‐Bed Times of Nursing Home Residents.” Journal of the American Geriatrics Society 52, no. 6: 931–938. 10.1111/j.1532-5415.2004.52260.x. 15161457

[opn70015-bib-0008] Bekdemir, A. , and N. Ilhan . 2019. “Predictors of Caregiver Burden in Caregivers of Bedridden Patients.” Journal of Nursing Research 27, no. 3: e24. 10.1097/jnr.0000000000000297. PMC6553964 30431539

[opn70015-bib-0009] Berger, B. , and S. Reuther . 2022. “Bettlägerigkeit–Das Bett Im Kopf Oder Heute Schon Die Weiße Decke Beobachtet?” In Förderung und Erhaltung der Mobilität in der Pflege alter Menschen: Empfehlungen für die Praxis, edited by B. Berger , F. Graeb , G. Essig , P. Reiber , and R. Wolke , 290–303. Kohlhammer.

[opn70015-bib-0010] Bruno, S. , M. José , S. Filomena , C. Vätor , M. Demetrio , and B. Karolina . 2016. “The Conceptual Design of a Mechatronic System to Handle Bedridden Elderly Individuals.” Sensors 16, no. 5: 725. 10.3390/s16050725. 27213383 PMC4883416

[opn70015-bib-0011] Campos, J. S. , A. C. Y. D. Anjos , S. B. D. C. Neto , and R. S. Peres . 2021. “Grieves and Struggles of Family Caregivers Providing Care for Bedridden Elderly Patients Affected by Chronic Degenerative Diseases.” Investigation Education Enfermeria 39, no. 2: e09. 10.17533/udea.iee.v39n2e09. PMC8253529 34214286

[opn70015-bib-0012] DNQP . 2014. “Expertenstandard nach § 113a SGB XI ‘Erhaltung und Förderung der Mobilität in der Pflege’.”

[opn70015-bib-0014] English, K. L. , and D. Paddon‐Jones . 2010. “Protecting Muscle Mass and Function in Older Adults During Bed Rest.” Current Opinion in Clinical Nutrition and Metabolic Care 13, no. 1: 34–39. 10.1097/MCO.0b013e328333aa66. 19898232 PMC3276215

[opn70015-bib-0015] Fletcher, K. 2005. “Immobility: Geriatric Self‐Learning Module.” Medical‐Surgical Nursing 14, no. 1: 35–37. 15779738

[opn70015-bib-0016] Fox, M. T. , S. Sidani , and D. Brooks . 2009. “Perceptions of Bed Days for Individuals With Chronic Illness in Extended Care Facilities.” Research in Nursing & Health 32, no. 3: 335–344. 10.1002/nur.20318. 19195038

[opn70015-bib-0017] Fox, M. T. , S. Sidani , and D. Brooks . 2010a. “Differences in Sleep Complaints in Adults With Varying Levels of Bed Days Residing in Extended Care Facilities for Chronic Disease Management.” Clinical Nursing Research 19, no. 2: 181–202. 10.1177/1054773810365957. 20435787

[opn70015-bib-0018] Fox, M. T. , S. Sidani , and D. Brooks . 2010b. “The Relationship Between Bed Rest and Sitting Orthostatic Intolerance in Adults Residing in Chronic Care Facilities.” Journal of Nursing and Healthcare of Chronic Illness 2, no. 3: 187–196. 10.1111/j.1752-9824.2010.01058.x.

[opn70015-bib-0019] Freiberger, E. , C. C. Sieber , and R. Kob . 2020. “Mobility in Older Community‐Dwelling Persons: A Narrative Review.” Frontiers in Physiology 11: 881. 10.3389/fphys.2020.00881. 33041836 PMC7522521

[opn70015-bib-0020] Futamura, M. , J. Sugama , M. Okuwa , H. Sanada , and K. Tabata . 2008. “Evaluation of Comfort in Bedridden Older Adults Using an Air‐Cell Mattress With an Automated Turning Function: Measurement of Parasympathetic Activity During Night Sleep.” Journal of Gerontological Nursing 34, no. 12: 20–26. 10.3928/00989134-20081201-09. 19113000

[opn70015-bib-0021] Gatt, M. E. , O. Paltiel , and M. Bursztyn . 2004. “Is Prolonged Immobilization a Risk Factor for Symptomatic Venous Thromboembolism in Elderly Bedridden Patients? Results of a Historical‐Cohort Study.” Thrombosis and Haemostasis 91, no. 3: 538–543. 10.1160/TH03-07-0481. 14983230

[opn70015-bib-0022] Gill, T. M. , H. Allore , and Z. Guo . 2004. “The Deleterious Effects of Bed Rest Among Community‐Living Older Persons.” Journals of Gerontology. Series A, Biological Sciences and Medical Sciences 59, no. 7: 755–761. 10.1093/gerona/59.7.m755. 15304541

[opn70015-bib-0023] Gill, T. M. , H. G. Allore , E. A. Gahbauer , and L. Han . 2015. “Establishing a Hierarchy for the Two Components of Restricted Activity.” Journal of Gerontology. Series A, Biological Sciences and Medical Sciences 70, no. 7: 892–898. 10.1093/gerona/glu203. PMC4481688 25391532

[opn70015-bib-0024] Gill, T. M. , H. G. Allore , E. A. Gahbauer , and T. E. Murphy . 2018. “Burden of Restricted Activity and Associated Symptoms and Problems in Late Life and at the End of Life.” Journal of the American Geriatrics Society 66, no. 12: 2282–2288. 10.1111/jgs.15566. 30277571 PMC6607906

[opn70015-bib-0025] Gill, T. M. , E. A. Gahbauer , L. Leo‐Summers , and T. E. Murphy . 2019. “Taking to Bed at the End of Life.” Journal of the American Geriatrics Society 67, no. 6: 1248–1252. 10.1111/jgs.15822. 30829402 PMC6986379

[opn70015-bib-0026] Guedes, L. P. C. M. , M. L. C. de Oliveira , and G. de Azevedo Carvalho . 2018. “Deleterious Effects of Prolonged Bed Rest on the Body Systems of the Elderly—A Review.” Revista Brasileira De Geriatria E Gerontologia 21, no. 4: 499–506. 10.1590/1981-22562018021.170167.

[opn70015-bib-0027] Hampton, S. 2011. “Practical Skin Care for People Who Are Bed‐Bound.” Nursing & Residential Care 13, no. 3: 132–134. http://www.redi‐bw.de/db/ebsco.php/search.ebscohost.com/login.aspx%3fdirect%3dtrue%26db%3dcin20%26AN%3d104649873%26site%3dehost‐live.

[opn70015-bib-0028] Hirakawa, Y. , Y. Masuda , T. Kimata , K. Uemura , M. Kuzuya , and A. Iguchi . 2005. “Effects of Home Massage Rehabilitation Therapy for the Bed‐Ridden Elderly: A Pilot Trial With a Three‐Month Follow‐Up.” Clinical Rehabilitation 19, no. 1: 20–27. 10.1191/0269215505cr795oa. 15704505

[opn70015-bib-0029] Ikezoe, T. , N. Mori , M. Nakamura , and N. Ichihashi . 2012. “Effects of Age and Inactivity due to Prolonged Bed Rest on Atrophy of Trunk Muscles.” Nursing & Residential Care 112, no. 1: 43–48. 10.1007/s00421-011-1952-x. 21472438

[opn70015-bib-0030] Imai, K. 1998. “Bed‐Ridden Elderly in Japan: Social Progress and Care for the Elderly.” International Journal of Aging and Human Development 46, no. 2: 157–170. 10.2190/HYAW-JPW6-633U-6HJE. 9572354

[opn70015-bib-0031] Izutsu, T. , T. Matsui , T. Satoh , T. Tsuji , and H. Sasaki . 1998. “Effect of Rolling Bed on Decubitus in Bedridden Nursing Home Patients.” Tohoku Journal of Experimental Medicine 184, no. 2: 153–157. 10.1620/tjem.184.153. 9605022

[opn70015-bib-0032] Kosaka, Y. , T. Nakagawa‐Satoh , T. Ohrui , M. Fujii , H. Arai , and H. Sasaki [Hidetada] . 2012. “Survival Period After Tube Feeding in Bedridden Older Patients.” Geriatrics & Gerontology International 12, no. 2: 317–321. 10.1111/j.1447-0594.2011.00805.x. 22239729

[opn70015-bib-0033] Levac, D. , H. Colquhoun , and K. K. O'Brien . 2010. “Scoping Studies: Advancing the Methodology.” Implementation Science 5: 69. 10.1186/1748-5908-5-69. 20854677 PMC2954944

[opn70015-bib-0034] Mamom, J. , and H. Daovisan . 2022. “Listening to Caregivers' Voices: The Informal Family Caregiver Burden of Caring for Chronically Ill Bedridden Elderly Patients.” International Journal of Environmental Research and Public Health 19, no. 1: 567. 10.3390/ijerph19010567. 35010827 PMC8744801

[opn70015-bib-0035] Okuwa, M. , H. Sanada , J. Sugama , et al. 2006. “A Prospective Cohort Study of Lower‐Extremity Pressure Ulcer Risk Among Bedfast Older Adults.” Advances in Skin & Wound Care 19, no. 7: 391–397. 10.1097/00129334-200609000-00017. 16943708

[opn70015-bib-0036] Peters, M. D. J. , C. Marnie , A. C. Tricco , et al. 2020. “Updated Methodological Guidance for the Conduct of Scoping Reviews.” JBI Evidence Synthesis 18, no. 10: 2119–2126. 10.11124/JBIES-20-00167. 33038124

[opn70015-bib-0037] Pinero de Planza, M. A. , A. Beleigoli , A. Mudd , et al. 2021. “Not Well Enough to Attend Appointments: Telehealth Versus Health Marginalisation…Digital Health Institute Summit, November 5–25, 2020.” Studies in Health Technology and Informatics 276: 72–79. 10.3233/SHTI210013.

[opn70015-bib-0038] Reuther, S. 2014. “Mobilitätsbeeinflussende Faktoren bei Bewohnern der Stationären Altenhilfe in Deutschland.” Pflege Und Gesellschaft 19, no. 2: 124–138.

[opn70015-bib-0039] Sanada, H. 2001. “Current Issues in Pressure Ulcer Management of Bedfast Elderly in Japan.” Journal of Tissue Viability 11, no. 1: 35–36. http://www.redi‐bw.de/db/ebsco.php/search.ebscohost.com/login.aspx%3fdirect%3dtrue%26db%3dcin20%26AN%3d106936314%26site%3dehost‐live. 11949309 10.1016/s0965-206x(01)80016-4

[opn70015-bib-0040] Santosa, A. , N. Puspitasari , and N. Isnaini . 2020. “A Path Analysis Study of Factors Influencing Decubitus in a Geriatric Nursing Home: A Preliminary Study.” Family Medicine and Primary Care Review 22, no. 1: 67–70. 10.5114/fmpcr.2020.92508.

[opn70015-bib-0041] Sato, S. , S. Demura , F. Goshi , M. Minami , H. Kobayashi , and Y. Nagasawa . 2001. “Utility of ADL Index for Partially Dependent Older People: Discriminating the Functional Level of an Older Population.” Journal of Physiological Anthropology and Applied Human Science 20, no. 6: 321–326. 10.2114/jpa.20.321. 11840683

[opn70015-bib-0042] Schirghuber, J. , and B. Schrems . 2018. “Ortsfixierung und Bettlägerigkeit im Kontext von Gebundenheit (Boundedness).” Pflege 31, no. 2: 87–99. 29375003 10.1024/1012-5302/a000606

[opn70015-bib-0043] Schirghuber, J. , and B. Schrems . 2021. “The Burden of Boundedness and the Implication for Nursing: A Scoping Review.” Nursing Forum 56, no. 4: 950–970. 10.1111/nuf.12637. 34312866 PMC9290579

[opn70015-bib-0044] Schirghuber, J. , and B. Schrems . 2023. “Being Wheelchair‐Bound and Being Bedridden: Two Concept Analyses.” Nursing Open 10, no. 4: 2075–2087. 10.1002/nop2.1455. 36336822 PMC10006658

[opn70015-bib-0045] Schrank, S. , A. Zegelin , and H. Mayer . 2013. “Prävalenzerhebung zur Bettlägerigkeit und Ortsfixierung.” Pflege 16, no. 4: 230–238.

[opn70015-bib-0046] Silva, L. P. , C. Fortaleza , N. B. Teixeira , L. Silva , C. D. de Angelis , and M. D. L. Ribeiro de Souza da Cunha . 2022. “Molecular Epidemiology of *Staphylococcus aureus* and MRSA in Bedridden Patients and Residents of Long‐Term Care Facilities.” Antibiotics 11, no. 11: 1526. 10.3390/antibiotics11111526. 36358181 PMC9686811

[opn70015-bib-0047] Taylor, D. H. , and H. Hoenig . 2004. “The Effect of Equipment Usage and Residual Task Difficulty on Use of Personal Assistance, Days in Bed, and Nursing Home Placement.” Journal of the American Geriatrics Society 52, no. 1: 72–79. 10.1111/j.1532-5415.2004.52013.x. 14687318

[opn70015-bib-0048] Tricco, A. C. , E. Lillie , W. Zarin , et al. 2018. “Prisma Extension for Scoping Reviews (PRISMA‐ScR): Checklist and Explanation.” Annals of Internal Medicine 169, no. 7: 467–473. 10.7326/M18-0850. 30178033

[opn70015-bib-0049] Tsuchihashi, T. , Y. Kawakami , T. Imamura , and I. Abe . 2002. “Reproducibility of Blood Pressure Variation in Older Ambulatory and Bedridden Subjects.” Journal of the American Geriatrics Society 50, no. 6: 1069–1074. 10.1046/j.1532-5415.2002.50262.x. 12110067

[opn70015-bib-0050] Vähäkangas, P. , A. Noro , H. Finne‐Soveri , and M. Björkgren . 2008. “Association Between Rehabilitation Care Practices and Care Quality in Long‐Term Care Facilities.” Journal of Nursing Care Quality 23, no. 2: 155–161. 10.1097/01.NCQ.0000313765.71772.66. 18344782

[opn70015-bib-0051] Wall, B. T. , M. L. Dirks , and L. van Loon . 2013. “Skeletal Muscle Atrophy During Short‐Term Disuse: Implications for Age‐Related Sarcopenia.” Ageing Research Reviews 12, no. 4: 898–906. 10.1016/j.arr.2013.07.003. 23948422

[opn70015-bib-0052] Walsh, K. , J. Roberts , and G. Bennett . 1999. “Mobility in Old Age.” Gerodontology 16, no. 2: 69–74. 10.1111/j.1741-2358.1999.00069.x. 10825844

[opn70015-bib-0053] Wick, J. Y. 2010. “Bed Rest: It May Not Be Such a Good Idea.” Consultant Pharmacist 25, no. 1: 59–62. 10.4140/TCP.n.2010.59. 20211817

[opn70015-bib-0054] Zegelin, A. 2005. “Tied Down—The Process of Becoming Bedridden Through Gradual Local Confinement.” Pflege 18, no. 5: 281–288. 10.1024/1012-5302.18.5.281. 16281892

[opn70015-bib-0055] Zegelin, A. 2008. “'Tied Down'—The Process of Becoming Bedridden Through Gradual Local Confinement.” Journal of Clinical Nursing 17, no. 17: 2294–2301. 10.1111/j.1365-2702.2007.02261.x. 18498343

[opn70015-bib-0056] Zegelin, A. 2010. Festgenagelt Sein. Verlag ‐ Hans Huber.

