# Supplementary material for: Becoming Bedridden and Being Bedridden: Implications for Nursing and Care for Older People in Long‐Term Care: A Scoping Review
Source: Int J Older People Nurs. 2025 Feb 13;20(2):e70015. doi: 10.1111/opn.70015 (PMC11823601; doi:10.1111/opn.70015)
Supplement: Supplementary file 1 — Data S1 [file OPN-20-e70015-s001.docx]

| **Category –** **“Process of Becoming Bedridden” (16)** | | | | | |
| --- | --- | --- | --- | --- | --- |
| **Author** | **Objective** | **Study Design** | **n** | **Setting** | **Country** |
| Bates-Jensen et al., 2004a | Comparing upper and lower quartile nursing homes on bedfast prevalence, resident activity, mobility care, and functional measures. | Cohort study | 451 | Nursing home | U.S. |
| Bates-Jensen et al., 2004b | Examining the effect of staffing level on the time observed in bed during the daytime in nursing-home residents. | Descriptive cross-sectional study | 882 | Nursing home | U.S. |
| Fox et al., 2009 | Exploring patient perceptions of bed days in extended inpatient services for chronic illness management. | Prospective cohort study | 46 | Care facilities for chronic disease management | Canada |
| Gill et al., 2004 | Examining the association between episodes of bed rest and functional decline was evaluated over an 18-month period. | Prospective cohort study | 680 | Community & home care | U.S. |
| Gill et al., 2015 | Assessing the impact of bed rest vs. reduced activity due to illness/injury. | Prospective cohort study | 754 | Community and home care | U.S. |
| Gill et al., 2018 | Comparing end-of-life activity^[[1]](#footnote-1)^ restrictions and associated symptoms in the last versus the previous six months. | Prospective cohort study | 737 | Community and home care | U.S. |
| Gill et al., 2019 | Assessing the time course of ‘bed rest’ at the end of life and examining variations based on age, sex, and cause of death. | Prospective longitudinal study | 651 | Community and home care | U.S. |
| Sato et al., 2001 | Clarifying the meaning of the evaluation of the ADL Index and examining how the ADL items are useful in determining functional level. | Cross-sectional study | 929 | Long-term-care facility | Japan |
| Schirghuber & Schrems, 2018 | Providing the basis for a conceptual definition. The current state of German and English usage of the terms ‘local confinement’ and ‘bedridden’ are presented. | Concept analysis | - | All health and care facilities | Austria |
| Schirghuber & Schrems, 2021b | Analyzing the concept and development of a conceptual definition of homebound. | Concept analysis | - | All health and care facilities | Austria |
| Schirghuber & Schrems, 2021a | Exploring the burden of location-boundness and its implications for nursing. | Scoping review | - | All health and care facilities | Austria |
| Schirghuber et al., 2022 | Validating and differentiating the location-bound model through a coordination process with nursing experts to develop evidence-based nursing diagnoses and interventions to prevent and reduce location-boundness. | Delphi study | - | All health and care facilities | Austria |
| Schirghuber & Schrems, 2023 | Analyzing the concepts and development of a conceptual definition of being wheelchair-bound and being bedridden. | Concept analysis |  | All health and care facilities | Austria |
| Schrank et al., 2013 | Determining the prevalence of bedriddenness and local confinement. | Cross-sectional study | 3054 | Nursing home | Austria |
| Zegelin, 2005 | Raising awareness of the process of becoming bedridden, creating knowledge about the different causes and types of being bedridden, and describing the factors that influence becoming bedridden. | Grounded theory | 32 | Nursing home and home care | Germany |
| Zegelin, 2008 | Gaining knowledge about the development of being confined to bed. | Grounded theory | 32 | Nursing home & homecare | Germany |

Table 1 Category - Process of Becoming Bedridden

Restricted activity, defined as staying in bed for at least half a day or cutting down on one’s usual activities because of illness, injury, or other problems,1 is an important source of disability and functional decline in older people.

| **Category – “Care of Bedridden Person” (14)** | | | | | |
| --- | --- | --- | --- | --- | --- |
| **Author** | **Objective** | **Study Design** | **n** | **Setting** | **Country** |
| Abarca et al., 2018 | Exploring caregiver perceptions on using technology for volunteer respite care for bedridden elders. | Ethnographic study | 10 | Community and home care | Chile |
| Bains & Minhas, 2011 | Ascertaining how the caregivers provide home-based care for adult bedridden patients. | Descriptive cross-sectional study | 305 | Community and home care | India |
| Bekdemir & Ilhan, 2019 | Determining how caregivers provide home-based care for adult bedridden patients. | Observational study | 312 | Community and home care | U.S. |
| Bruno et al., 2016 | Presenting an approach for supporting caregivers when moving and repositioning bedridden older adults people in home settings using a mechatronic system inspired by industrial conveyors. | Design research | k. A. | Home care | Portugal |
| Campos et al., 2021 | Understanding grieves and struggles of family caregivers caring for bedridden older patients affected by chronic degenerative diseases. | Observational study | 10 | Community and home care | Brazil |
| Futamura et al., 2008 | Evaluating the degree of comfort in bedridden older adults using an air-cell mattress with an automated turning mechanism. | Quasi-experimental study | 10 | Long-term-care facility | Japan |
| Hirakawa et al., 2005 | Assessing the effects of home massage rehabilitation therapy on bedridden older adults. | RCT | 40 | Community and home care | Japan |
| Imai, 1998 | Exploring the impact of the increasing number of bedridden older adult individuals on social problems related to economic development and population changes. | Comparative epidemiological study | 64,7 Mio. | All health and care facilities | Japan |
| Izutsu et al., 1998 | Developing a bed with a rolling air cushion that turns the patient to a 15° inclined lateral position, equipped with an inflating ripple mattress and a longitudinally aligned air inflatable tube. | RCT | 31 | Long-term-care facility | Japan |
| Kosaka et al., 2012 | Studying survival periods after tube feeding in bedridden older patients. | Prospective cohort study | 163 | Long-term-care facility | Japan |
| Mamom & Daovisan, 2022 | Addressing the gap in caregiver theory for chronically ill, bedridden older patients in palliative care. | Structural modelling approach | 30 | Community & homecare | Thailand |
| Pinero de Planza, M.A. et al., 2021 | Illustrating the health exclusion and marginalization of frail, homebound, and bedridden persons. | Consumer Research | 164 | Community & homecare | Australia |
| Tsuchihashi et al., 2002 | Investigating the influence of ambulation on circadian blood pressure variation reproducibility among older bedridden nursing-home residents. | Cohort study | 37 | Nursing home | Japan |
| Walsh et al., 1999 | Discussing common causes and complications of immobility, medical management strategies for older patients, and the challenges that immobile patients may pose to dental practitioners. | Overview-continuing education | - | All health and care facilities | UK |

Table 2 Category - Care of Bedridden Person

| **Category – “****Adverse Outcomes & Consequences of Bedriddenness and Their Treatment and Prevention” (9)** | | | | | |
| --- | --- | --- | --- | --- | --- |
| **Author** | **Objective** | **Study Design** | **n** | **Setting** | **Country** |
| Fox et al., 2010a | Investigating perceived insomnia and daytime sleepiness in adults with varying bed days. | Cohort study | 67 | Care facilities for chronic disease management | Canada |
| Fox et al., 2010b | Examining orthostatic intolerance and bed rest in older adults who are long-term care residents. | Prospective cohort study | 65 | Long-term-care facility | Canada |
| Gatt et al., 2004 | Assessing whether extended immobilization (i.e., over three months) increases the risk for clinically apparent venous thromboembolic events. | Retrospective cohort study | 471 | Nursing home | Israel |
| Hampton, 2011 | Practical advice for identifying, treating, and preventing skin problems in the group of bedridden persons. | Overview-continuing education | - | Nursing home | UK |
| Ikezoe et al., 2012 | Investigating the effects of age and inactivity due to being chronically bedridden on atrophy of trunk muscles. | Case–control study | 41^[[2]](#footnote-2)^ | Nursing home and long-term-care facility | Japan |
| Okuwa et al., 2006 | Estimating the incidence and identifying risk factors for lower extremity pressure ulcers in bedfast older adult patients. | Prospective cohort study | 259 | Long-term-care facility | Japan |
| Santosa et al., 2020 | Analyzing the effect of nutritional status and bed rest on pressure, humidity, and friction and incidence of decubitus. | Case–control study | 60 | Nursing home | Indonesia |
| Silva et al., 2022 | Identifying the prevalence and factors associated with nasal, oral and rectal carriage of Staphylococcus aureus and MRSA in bedridden patients and residents of long-term care facilities for older adults. | Cross-sectional study | 226 | Long-term-care facility | Brazil |
| Wick, 2010 | Describing and consequences of bed rest and prevention strategies. Exploring the cascade of dependency and risk factors that may arise from prolonged immobility. | Overview-continuing education | - | Nursing home | U.S. |

Table 3 Category - Adverse Outcomes & Consequences of Bedriddenness and Their Treatment and Prevention

| **Category – “****Prevention of Bedriddenness” (8)** | | | | | | |
| --- | --- | --- | --- | --- | --- | --- |
| **Author** | **Objective** | **Study Design** | **n** | **Setting** | **Country** |  |
| Alexander et al., 2000 | Investigating the ability and time taken by older adults with ADL impairments to rise from a bed or chair under various conditions. | Experimental study | 116 | Community and home care | U.S. | |
| Arentson-Lantz et al., 2019 | Examining whether 2000 daily steps, with minimal other activities, can mitigate inactivity’s catabolic stress and maintain muscle health. | Experimental study | 17 | All health and care facilities | U.S. | |
| English & Paddon-Jones, 2010 | Addressing muscle and functional losses in bedridden older adults and offering prevention and rehabilitation strategies. | Literature review | - | - | U.S. | |
| Fletcher, 2005 | Promoting awareness of immobility hazards among older adults and enhancing mobility intervention strategies. | Continuing Education Module | - | Long-term-care facility | U.S. | |
| Reuther, 2014 | Identifying factors affecting the mobility of residents in nursing homes. | Case analyses | 50 | Nursing home | Germany | |
| Taylor & Hoenig, 2004 | Determining whether residual difficulty in functioning despite equipment use is linked with increased use of personal assistance. | Longitudinal cohort study | 8222 | Nursing home and home care | U.S. | |
| Vähäkangas et al., 2008 | Analyzing the association between rehabilitation care practices and quality outcomes and representing unit-level associations between rehabilitation nursing and quality outcomes. | Cohort Study | 256 | Long-term-care facility | Finland | |
| Wall et al., 2013 | Examining muscle loss in older people during bed rest to develop interventions for sarcopenia prevention. | Review | - | All health and care facilities | Netherlands | |

Table 4 Category - Prevention of Bedriddenness

References

Abarca, E., Campos, S., Herskovic, V., & Fuentes, C. (2018). Perceptions on technology for volunteer respite care for bedridden elders in Chile. *Int J Qual Stud Health Well-Being*, *13*(1), 1422663. https://doi.org/10.1080/17482631.2017.1422663

Alexander, N. B., Galecki, A. T., Nyquist, L. V., Hofmeyer, M. R., Grunawalt, J. C., Grenier, M. L., & Medell, J. L. (2000). Chair and bed rise performance in ADL-impaired congregate housing residents. *J Am Geriatr Soc*, *48*(5), 526–533. https://doi.org/10.1111/j.1532-5415.2000.tb04999.x

Arentson-Lantz, E., Galvan E, Wacher A, Fry CS, & Paddon-Jones D (2019). 2,000Â Steps/Day Does Not Fully Protect Skeletal Muscle Health in Older Adults During Bed Rest. *J Aging Phys Act*, *27*(2), 191–197. https://doi.org/10.1123/japa.2018-0093

Bains, P., & Minhas, A. S. (2011). Profile of Home-based Caregivers of Bedridden Patients in North India. *Indian Journal of Community Medicine*, *36*(2), 114–119. http://www.redi-bw.de/db/ebsco.php/search.ebscohost.com/login.aspx%3fdirect%3dtrue%26db%3dcin20%26AN%3d104689123%26site%3dehost-live

Bates-Jensen, B. M., Alessi, C. A., Cadogan, M., Levy-Storms, L., Jorge, J., Yoshii, J., Al-Samarrai, N. R., & Schnelle, J. F. (2004a). The Minimum Data Set bedfast quality indicator: differences among nursing homes. *Nurs Res*, *53*(4), 260–272. https://doi.org/10.1097/00006199-200407000-00009

Bates-Jensen, B. M., Schnelle, J. F., Alessi, C. A., Al-Samarrai, N. R., & Levy-Storms, L. (2004b). The effects of staffing on in-bed times of nursing home residents. *J Am Geriatr Soc*, *52*(6), 931–938. https://doi.org/10.1111/j.1532-5415.2004.52260.x

Bekdemir, A., & Ilhan, N. (2019). Predictors of Caregiver Burden in Caregivers of Bedridden Patients. *Journal of Nursing Research (Lippincott Williams & Wilkins)*, *27*(3), e24-e24. https://doi.org/10.1097/jnr.0000000000000297

Bruno, S., José, M., Filomena, S., Vä­tor, C., Demetrio, M., & Karolina, B. (2016). The Conceptual Design of a Mechatronic System to Handle Bedridden Elderly Individuals. *Sensors (Basel)*, *16*(5). https://doi.org/10.3390/s16050725

Campos, J. S., Anjos, A. C. Y. D., Neto S. B. D. C., & Peres, R. S. (2021). Grieves and struggles of family caregivers providing care for bedridden elderly patients affected by chronic degenerative diseases. *Invest Educ Enferm*, *39*(2). https://doi.org/10.17533/udea.iee.v39n2e09

English, K. L., & Paddon-Jones, D. (2010). Protecting muscle mass and function in older adults during bed rest. *Curr Opin Clin Nutr Metab Care*, *13*(1), 34–39. https://doi.org/10.1097/MCO.0b013e328333aa66

Fletcher, K. (2005). Immobility: geriatric self-learning module. *Medsurg Nurs*, *14*(1), 35–37.

Fox, M. T., Sidani, S., & Brooks, D. (2009). Perceptions of bed days for individuals with chronic illness in extended care facilities. *Res Nurs Health*, *32*(3), 335–344. https://doi.org/10.1002/nur.20318

Fox, M. T., Sidani, S., & Brooks, D. (2010a). Differences in sleep complaints in adults with varying levels of bed days residing in extended care facilities for chronic disease management. *Clin Nurs Res*, *19*(2), 181–202. https://doi.org/10.1177/1054773810365957

Fox, M. T., Sidani, S., & Brooks, D. (2010b). The relationship between bed rest and sitting orthostatic intolerance in adults residing in chronic care facilities. *Journal of Nursing & Healthcare of Chronic Illnesses*, *2*(3), 187–196. https://doi.org/10.1111/j.1752-9824.2010.01058.x

Futamura, M., Sugama, J., Okuwa, M., Sanada, H., & Tabata, K. (2008). Evaluation of comfort in bedridden older adults using an air-cell mattress with an automated turning function: measurement of parasympathetic activity during night sleep. *J Gerontol Nurs*, *34*(12), 20–26. https://doi.org/10.3928/00989134-20081201-09

Gatt, M. E., Paltiel, O., & Bursztyn, M. (2004). Is prolonged immobilization a risk factor for symptomatic venous thromboembolism in elderly bedridden patients? Results of a historical-cohort study. *Thromb Haemost*, *91*(3), 538–543. https://doi.org/10.1160/TH03-07-0481

Gill, T. M., Allore, H. G., Gahbauer, E. A., & Han, L. (2015). Establishing a Hierarchy for the Two Components of Restricted Activity. *J Gerontol a Biol Sci Med Sci*, *70*(7), 892–898. https://doi.org/10.1093/gerona/glu203

Gill, T. M., Allore, H. G., Gahbauer, E. A., & Murphy, T. E. (2018). Burden of Restricted Activity and Associated Symptoms and Problems in Late Life and at the End of Life. *J Am Geriatr Soc*, *66*(12), 2282–2288. https://doi.org/10.1111/jgs.15566

Gill, T. M., Allore, H., & Guo, Z. (2004). The deleterious effects of bed rest among community-living older persons. *J Gerontol a Biol Sci Med Sci*, *59*(7), 755–761. https://doi.org/10.1093/gerona/59.7.m755

Gill, T. M., Gahbauer EA, Leo-Summers L, & Murphy TE (2019). Taking to Bed at the End of Life. *J Am Geriatr Soc*, *67*(6), 1248–1252. https://doi.org/10.1111/jgs.15822

Hampton, S. (2011). Practical skin care for people who are bed-bound. *Nursing & Residential Care*, *13*(3), 132–134. http://www.redi-bw.de/db/ebsco.php/search.ebscohost.com/login.aspx%3fdirect%3dtrue%26db%3dcin20%26AN%3d104649873%26site%3dehost-live

Hirakawa, Y., Masuda, Y., Kimata, T., Uemura, K., Kuzuya, M., & Iguchi, A. (2005). Effects of home massage rehabilitation therapy for the bed-ridden elderly: a pilot trial with a three-month follow-up. *Clin Rehabil*, *19*(1), 20–27. https://doi.org/10.1191/0269215505cr795oa

Ikezoe, T., Mori, N., Nakamura, M., & Ichihashi, N. (2012). Effects of age and inactivity due to prolonged bed rest on atrophy of trunk muscles. *Nursing & Residential Care*, *112*(1), 43–48. https://doi.org/10.1007/s00421-011-1952-x

Imai, K. (1998). Bed-ridden elderly in Japan: social progress and care for the elderly. *Int J Aging Hum Dev*, *46*(2), 157–170. https://doi.org/10.2190/HYAW-JPW6-633U-6HJE

Izutsu, T., Matsui, T., Satoh, T., Tsuji, T., & Sasaki, H [H.] (1998). Effect of rolling bed on decubitus in bedridden nursing home patients. *Tohoku J Exp Med*, *184*(2), 153–157. https://doi.org/10.1620/tjem.184.153

Kosaka, Y., Nakagawa-Satoh, T., Ohrui, T., Fujii, M., Arai, H., & Sasaki, H [Hidetada] (2012). Survival period after tube feeding in bedridden older patients. *Geriatrics & Gerontology International*, *12*(2), 317–321. https://doi.org/10.1111/j.1447-0594.2011.00805.x

Mamom, J., & Daovisan, H. (2022). Listening to Caregivers' Voices: The Informal Family Caregiver Burden of Caring for Chronically Ill Bedridden Elderly Patients. *Int J Environ Res Public Health*, *19*(1). https://doi.org/10.3390/ijerph19010567

Okuwa, M., Sanada, H., Sugama, J., Inagaki, M., Konya, C., Kitagawa, A., & Tabata, K. (2006). A prospective cohort study of lower-extremity pressure ulcer risk among bedfast older adults. *Adv Skin Wound Care*, *19*(7), 391–397. https://doi.org/10.1097/00129334-200609000-00017

Pinero de Planza, M.A., Beleigoli, A., Mudd, A., Tieu, M., McMillian, P., Lawless, M., Feo, R., & Archibald, M. & Kitson, A. (2021). Not Well Enough to Attend Appointments: Telehealth Versus Health Marginalisation. Digital Health Institute Summit, November 5-25, 2020. *Studies in Health Technology & Informatics, 276*, 72–79. https://doi.org/10.3233/SHTI210013

Reuther, S. (2014). Mobilitätsbeeinflussende Faktoren bei Bewohnern der stationären Altenhilfe in Deutschland. *Pflege Und Gesellschaft*, *19*(2), 124–138.

Santosa, A., Puspitasari, N., & Isnaini, N. (2020). A path analysis study of factors influencing decubitus in a geriatric nursing home: A preliminary study. *Fam Med Prim Care Rev*, *22*(1), 67–70. https://doi.org/10.5114/fmpcr.2020.92508

Sato, S., Demura S, Goshi F, Minami M, Kobayashi H, & Nagasawa Y (2001). Utility of ADL index for partially dependent older people: discriminating the functional level of an older population. *J Physiol Anthropol Appl Human Sci*, *20*(6), 321–326. https://doi.org/10.2114/jpa.20.321

Schirghuber, J., Köck-Hódi, S., & Schrems, B. (2022). „Nicht mehr raus können!“ Validität und Differenzierung der Konzepte Hausgebundenheit, Rollstuhlgebundenheit und Bettlägerigkeit: eine Delphi-Studie ["If you're no longer able to get out and about …" Validity and differentiation of the concepts of being homebound, wheelchair-bound and bedridden: A Delphi study]. *Zeitschrift fur Evidenz, Fortbildung und Qualitat im Gesundheitswesen*, *173*, 1–16. https://doi.org/10.1016/j.zefq.2022.07.003

Schirghuber, J., & Schrems, B. (2018). Ortsfixierung und Bettlägerigkeit im Kontext von Gebundenheit (boundedness). *Pflege*, *31*(2), 87–99.

Schirghuber, J., & Schrems, B. (2021a). The burden of boundedness and the implication for nursing: A scoping review. *Nursing Forum*, *56*(4), 950–970. https://doi.org/10.1111/nuf.12637

Schirghuber, J., & Schrems, B. (2021b). Homebound: A concept analysis. *Nursing Forum*, *56*(3), 742–751. https://doi.org/10.1111/nuf.12586

Schirghuber, J., & Schrems, B. (2023). Being wheelchair-bound and being bedridden: Two concept analyses. *Nursing Open*, *10*(4), 2075–2087. https://doi.org/10.1002/nop2.1455

Schrank, S., Zegelin, A., & Mayer, H. (2013). Prävalenzerhebung zur Bettlägerigkeit und Ortsfixierung. *Pflegewissenschaft*, *16*(4), 230–238.

Silva, L. P., Fortaleza, C., Teixeira, N. B., Silva, L., Angelis, C. D. de, & Ribeiro de Souza da Cunha, M.D.L. (2022). Molecular Epidemiology of Staphylococcus aureus and MRSA in Bedridden Patients and Residents of Long-Term Care Facilities. *Antibiotics*, *11*(11). https://doi.org/10.3390/antibiotics11111526

Taylor, D. H., & Hoenig, H. (2004). The effect of equipment usage and residual task difficulty on use of personal assistance, days in bed, and nursing home placement. *J Am Geriatr Soc*, *52*(1), 72–79. https://doi.org/10.1111/j.1532-5415.2004.52013.x

Tsuchihashi, T., Kawakami, Y., Imamura, T., & Abe, I. (2002). Reproducibility of blood pressure variation in older ambulatory and bedridden subjects. *J Am Geriatr Soc*, *50*(6), 1069–1074. https://doi.org/10.1046/j.1532-5415.2002.50262.x

Vähäkangas, P., Noro, A., Finne-Soveri, H., & Björkgren, M. (2008). Association between rehabilitation care practices and care quality in long-term care facilities. *J Nurs Care Qual*, *23*(2), 155–161. https://doi.org/10.1097/01.NCQ.0000313765.71772.66

Wall, B. T., Dirks, M. L., & van Loon, L. (2013). Skeletal muscle atrophy during short-term disuse: Implications for age-related sarcopenia. *Ageing Res. Rev.*, *12*(4), 898–906. https://doi.org/10.1016/j.arr.2013.07.003

Walsh, K., Roberts J., Bennett G. (1999). Mobility in old age. *Gerodontology*, *16*(2), 69–74. https://doi.org/10.1111/j.1741-2358.1999.00069.x

Wick, J. Y. (2010). Bed rest: it may not be such a good idea. *Consult Pharm*, *25*(1), 59–62. https://doi.org/10.4140/TCP.n.2010.59

Zegelin, A. (2005). "Tied down"--the process of becoming bedridden through gradual local confinement. *Pflege*, *18*(5), 281–288. https://doi.org/10.1024/1012-5302.18.5.281

Zegelin, A. (2008). 'Tied down'- the process of becoming bedridden through gradual local confinement. *J Clin Nurs*, *17*(17), 2294–2301. https://doi.org/10.1111/j.1365-2702.2007.02261.x

1. [↑](#footnote-ref-1)
2. **Note**: Only elderly women (aged 85.7 ± 5.5 and 87.8 ± 6) were considered. They were divided into a group of independent older women who were able to perform daily activities independently (n = 28), and a group of dependent older women who were chronically bedridden (n = 13) [↑](#footnote-ref-2)
