# Supplementary material for: Becoming Bedridden and Being Bedridden: Implications for Nursing and Care for Older People in Long‐Term Care: A Scoping Review
Source: Int J Older People Nurs. 2025 Feb 13;20(2):e70015. doi: 10.1111/opn.70015 (PMC11823601; doi:10.1111/opn.70015)
Supplement: Supplementary file 2 — Data S2 [file OPN-20-e70015-s002.docx]

Studies from databases/registers **(n = 1066)**

PubMed (n = 426)

LIVIO (n = 384)

Scopus (n = 143)

CINAHL (n = 106)

References from other sources **(n =7)**

**Identification**

Studies included in review **(47)**

References removed **(n = 416)**

Duplicates identified manually (n = 45)

Duplicates identified by Covidence (n = 371)

Studies screened **(n = 650)**

Studies excluded **(n = 401)**

Studies sought for retrieval **(n = 249)**

Studies not retrieved **(n = 0)**

**Screening**

Studies excluded **(n= 201)**

Not available (n = 3)

Wrong indication (n = 44)

Retracted Article (n = 3)

Wrong intervention (n = 24)

Wrong study design (n = 10)

Wrong patient population (n = 26)

Wrong Setting (n =62)

Summary of an included study (n = 1)

Identical article with different publication dates (n = 1) Not peer-reviewed (n=4)

Date before 1998 (n=25)

Studies assessed for eligibility **(n = 249)**

**Included**
