# Supplementary material for: Becoming Bedridden and Being Bedridden: Implications for Nursing and Care for Older People in Long‐Term Care: A Scoping Review
Source: Int J Older People Nurs. 2025 Feb 13;20(2):e70015. doi: 10.1111/opn.70015 (PMC11823601; doi:10.1111/opn.70015)
Supplement: Supplementary file 3 — Data S3 [file OPN-20-e70015-s003.docx]

| **Search Strings**  The strategy was developed by identifying key words from the research question, as well as by identifying additional keywords, meshterms through an exploratory search. | |
| --- | --- |
| **Databases** | **Search string** |
| **PubMed (2023/01)** | ((("care*"[All Fields] OR "Patient Care"[MeSH Terms] OR "nurs*"[All Fields] OR "Nursing"[MeSH Terms]) AND ("bedridden*"[Title] OR "Bedridden Persons"[MeSH Terms] OR "bedrest"[Title] OR "bed-rest"[Title] OR "bed-rest"[MeSH Terms] OR "bedbound*"[Title] OR "bed-bound*"[Title] OR "bed bound*"[Title] OR "bedfast"[Title] OR "bed-fast"[Title] OR "bed fast"[Title] OR ("bedridden*"[Title/Abstract] AND ("bedrest"[Title/Abstract] OR "bed-rest"[Title/Abstract] OR "bed rest"[Title/Abstract])))) OR (("bedridden*"[Title] OR "Bedridden Persons"[MeSH Terms] OR "bedrest"[Title] OR "bed-rest"[Title] OR "bed rest"[Title] OR "bed-rest"[MeSH Terms] OR "bedbound*"[Title] OR "bed-bound*"[Title] OR "bed bound*"[Title] OR "bedfast"[Title] OR "bed-fast"[Title] OR "bed fast"[Title] OR ("bedridden*"[Title] AND ("bedrest"[Title] OR "bed-rest"[Title] OR "bed rest"[Title]))) AND ("concept"[All Fields] OR "concepts"[All Fields] OR "definition*"[All Fields] OR "intervention"[All Fields]))) |
| **Filter** | - Language German or English - Aged 65+ or Aged 80 and over or aged |
| **CINAHL, LIVIO, SCOPUS (2023/04)** | The search strategy was adapted for each database to account for differences in terminology and indexing systems. The strategy was tailored to ensure comprehensive coverage of relevant literature across the following databases: CINAHL (04/2023), LIVIO (04/2023) and SCOPUS (04 /2023). |
| Studies published before 1998, as well as those focusing on general hospital settings, were excluded. This step narrowed the focus to long-term care settings, specifically those related to the care of older adults (see Supplement 2). | |
